# Short-term vs. overnight insemination: which one is better for patients with only one or two oocytes retrieved in IVF cycles?

**DOI:** 10.3389/fendo.2026.1647862

**Published:** 2026-02-03

**Authors:** Jinghua Chen, Zhenfang Liu, Jiali Cai, Xiaoming Jiang, Luxiang Pan, Liying Zhou, Kaijie Chen, Xiaolian Yang, Yurong Chen, Lanlan Liu, Jianzhi Ren

**Affiliations:** 1 Reproductive Medicine Center, Xiamen University Affiliated Chenggong Hospital, Xiamen, Fujian, China; 2 School of Medicine, Xiamen University, Xiamen, Fujian, China

**Keywords:** short-term insemination, overnight insemination, IVF-ET, normal fertilization rate, blastocyst formation rate, cumulative live birth rate

## Abstract

**Background:**

Compared with overnight insemination, it remains uncertain whether short-term insemination can improve outcomes in poor responders, and few studies have used cumulative live birth rate as the primary endpoint. The study aims to compare the effects of short-term insemination and overnight insemination on poor responders with one or two oocytes by evaluating cumulative live births.

**Methods:**

A retrospective study was conducted on 2,392 cycles, which were divided into the short-term insemination group (5 h) and the overnight insemination group (17 h) based on gametes’ incubation time. Basic characteristics and clinical outcomes were compared and analyzed using propensity score matching.

**Results:**

The short-term insemination group got more normal fertilized embryos than the overnight insemination group (*P* < 0.05). The cumulative live birth rates between the two groups were similar before matching (21.1% vs. 17.6%, *P* > 0.05) and after matching (20.7% vs 17.9%, *P* > 0.05). Multivariate logistic regression analysis confirmed that short-term insemination had no adverse effect on the cumulative live birth rate (CLBR). However, in the subgroup of patients who underwent extended culture of all embryos, comparing overnight to short-term insemination, the odds ratios (ORs) for CLBR were 0.40 (95% CI: 0.18–0.88) in the unmatched cohort and 0.39 (95% CI: 0.18–0.84) in the matched cohort.

**Conclusions:**

Our analysis suggests that for IVF cycles with a limited oocyte yield (1–2), short-term insemination does not adversely affect the CLBR. Furthermore, it may demonstrate a potential benefit for the subset of patients whose clinical pathway involves the extended culture of all embryos to the blastocyst stage.

## Introduction

In conventional *in vitro* fertilization (IVF), oocytes and sperm are typically co-incubated for 16–24 h before the removal of cumulus cells (1). However, concerns have been raised regarding the potential stress and adverse effects associated with this prolonged co-incubation period (2–4). Prolonged co-culture with sperm, cumulus cells, and other debris may produce high levels of reactive oxygen species (ROS) (5, 6), which might be detrimental to fertilization and subsequent embryonic development. Previous studies have shown that ROS produced by cumulus cells and sperm metabolism can affect embryo division and development and, thus, decrease embryo implantation (7, 8).

Earlier studies have shown that sperm–oocyte interaction takes place within 1 h of oocytes being exposed to sperm. Consequently, shortening the insemination duration could serve as a viable strategy to mitigate the potential adverse effects associated with the overnight *in vitro* co-incubation of gametes. The so-called “short-term insemination” is defined as an insemination procedure wherein oocytes are removed from the insemination medium following 1–6 h of exposure to spermatozoa (3). Incorporated with the early removal of cumulus cells, short-term insemination also allows early rescue intracytoplasmic sperm injection (ICSI), which may provide an early intervention option for unexpected fertilization failure during conventional IVF with notable success rates (9–11).

However, the pros and cons of short-term insemination remained debatable. A 2014 meta-analysis showed that short-term insemination could significantly improve implantation rate and clinical pregnancy rate compared with overnight insemination (12). Several recent studies also showed a potential benefit of short-term insemination on implantation rates and clinical pregnancy rates (13, 14). Conversely, other reports have noted similar or even less favorable outcomes with short-term insemination when compared to overnight insemination (15, 16). Moreover, several researchers highlighted that short-term insemination is associated with an increased number of multiple pronuclei (MPN), which may further affect embryo availability and thus affect the chance of subsequent cycles (15, 17).

For poor responders, embryo availability and MPN remain significant concerns. A reduced normal fertilization rate may lead to no embryo transfer in poor responders with only one or two oocytes. Additionally, poor responders are less likely to benefit from the rescue ICSI opportunity that accompanies early cumulus cell removal (18, 19). It may also be said that short-term insemination is less suitable for poor responders. On the other hand, however, with extremely low oocyte yield, poor responders may be more vulnerable to the stress generated during the treatment procedures, including those generated during insemination. As such, the pros and cons of short-term insemination may both have a more profound effect on poor responders than on normal responders.

Few studies compared the effect of short-term insemination and overnight insemination on poor responders. Additionally, most previous reports focused on outcomes of fresh embryo transfer cycles (3, 15), and this may introduce bias by excluding patients undergoing freeze-all or having no embryos for transfer, both of which are common scenarios in the treatment of poor responders. In contrast, cumulative live birth (CLBR) following a complete cycle, considering all possible chances of embryo transfer, provides a more comprehensive evaluation of clinical and laboratory outcomes. Therefore, the present study aims to compare the effects of short-term insemination and overnight insemination on poor responders with only one or two oocytes retrieved, using cumulative live births as the primary evaluation endpoint, and cycle cancellation rate and normal fertilization rate were used as secondary outcomes.

## Materials and methods

### Study subjects

A retrospective analysis was performed on patients who underwent IVF treatment at the Reproductive Medicine Center of Xiamen University Affiliated Chenggong Hospital, from January 2013 to December 2022. These patients were divided into the short-term insemination group (5 h) and the overnight insemination group (17 h) based on the differences in gamete incubation times. (The assignment of the patients was due to a shift in our policy. Before 2020, short-term insemination was used for all IVF-indicated patients regardless of the ovarian response. Since 2021, however, the overnight insemination protocol has been adopted specifically for patients with only one or two retrieved oocytes. This policy adjustment was made because there is a potential risk in determining oocyte fertilization by excluding the second polar body; moreover, patients with just one or two oocytes are unlikely to benefit from rescue ICSI. All patients who underwent IVF treatment with only one or two oocytes were not considered for rescue ICSI in our center.) The inclusion criteria were as follows: 1) patients who had only one or two oocytes retrieved during their IVF treatment; and 2) all cycles included must have either resulted in a live birth or had no remaining frozen embryos. We excluded 198 patients with a history of intrauterine adhesion, 16 patients with abnormal uterine anatomy, and 59 patients with previous cesarean scar diverticulum (PCSD).

Institutional review board approval for this study was obtained from Xiamen University Affiliated Chenggong Hospital in accordance with the Declaration of Helsinki. Informed consent was not necessary because this retrospective research was based on non-identifiable records.

### Laboratory procedures and embryo assessment

Different ovarian stimulation protocols were selected according to the patient’s individual clinical conditions, and follicular development was monitored by transvaginal ultrasound and hormone levels. When the diameter of one dominant follicle reached ≥18 mm or the diameter of two dominant follicles reached ≥17 mm, human chorionic gonadotropin (hCG) at a dose of 10,000 U was injected intramuscularly. After 36–38 h post-hCG administration, oocyte retrieval was performed by transvaginal ultrasound, and follicles were aspirated by a 17-G needle (Cook Medical, IN, USA).

On the day of oocyte pick-up, semen samples were collected in sterile containers and evaluated for sperm density, motility, and morphology in accordance with the World Health Organization (WHO) criteria (20). Sperm was prepared by density gradient centrifugation combined with the swim-up method. Patients with more than 2 million motile spermatozoa were recommended for IVF (21). Sperm concentration was adjusted to 2 × 10^5^/mL for insemination with the cumulus–oocyte complex and incubated in an incubator (C200, Labotect, Göttingen, Germany) at 37°C, 6% CO_2_, and 5% O_2_ in a humidified atmosphere.

The cumulus cells around the oocytes were mechanically removed by a 140-μm pipette under an inverted microscope at 5 h (short-term insemination group) or 17 h (overnight insemination group) after fertilization in the well. Fertilization was judged according to whether the pronucleus appeared at 17 ± 1 h after insemination. Normal fertilization was defined as the observation of two pronuclei (2PN), while multiple pronuclei was defined as the presence of ≥3 pronuclei (3PN). Embryos were cultured in cleavage medium (K-SICM, Cook) on days 1–3 after fertilization and then in blastocyst medium (K-SIBM, Cook) on days 4–7. Day 3 embryo assessment and grading system was based on the number of embryo blastomeres, fragmentation, and symmetry. According to the Istanbul Consensus embryo evaluation criteria (22), 2PN embryos on day 3 were evaluated and classified into four different grades: grade 1 and grade 2 embryos were considered high-quality embryos; grade 1, grade 2, and grade 3 embryos were considered available embryos, while grade 4 embryos, arrested embryos, and embryos with all blastomere degenerated or lysed were typically discarded. For blastocyst assessment, we used the Gardner grading system (23), and high-quality blastocysts h were defined as those with a score of ≥4 BB. Blastocysts with poor morphological score (≤ CC) or low expansion grade (grades 1–2) were neither frozen nor transplanted.

A vitrification protocol was used for embryo cryopreservation, which employed 15% dimethyl sulfoxide, 15% ethylene glycol, and 0.6 M sucrose as cryoprotectants. For embryo thawing, embryos were directly immersed in a thawing solution (TS) with a concentration of 1 M sucrose at 37°C for 1 min. Subsequently, they were sequentially incubated in the following solutions for 3 min: 0.5 M sucrose, 0.25 M sucrose, and sucrose-free TS, and then embryos were transferred to blastocyst culture medium for subsequent normal culture.

In either fresh embryo or frozen embryo transfer cycles, embryo transfer was scheduled on day 3 for cleavage-stage embryos and on day 5 for blastocyst transfer. Embryo transfer was performed using a Cook catheter (Cook, IN, USA) under trans-abdominal ultrasound guidance. Serum β-hCG was measured 14 days after embryo transfer to determine pregnancy status. Clinical pregnancy was defined by the presence of a gestational sac on B-ultrasound, and live birth was defined as a live birth of a fetus.

CLBR, which is defined as the first live birth after using all fresh and frozen embryos from a complete oocyte retrieval cycle (24), is the primary outcome of interest. A complete cycle was defined as a cycle achieving a live birth or having all embryos transferred. The secondary outcomes were cycle cancellation, fertilization, and embryo development.

### Statistical analysis

The propensity score (PS) matching method was used to minimize the effect of confounders and covariates; outcomes were evaluated in both groups before and after matching. The selection of covariates was based on experience and existing research knowledge, with the guidance of a directed acyclic graph with DAGitty software (**Supplementary Figure S1**).

For PS matching, the covariates included female age, history of spontaneous miscarriage, parity, oocyte pick-up (OPU) order, diagnosis of endometriosis or polycystic ovary syndrome, the duration of infertility, female BMI, basal FSH, basal LH, antral follicle count (AFC), male age, male BMI, sperm normal morphology rate, TMC (total motile sperm count), ovarian stimulation protocol, gonadotropin starting dose, oocyte yield, and extended embryo culture. Propensity score matching was performed between the two groups at a 2:1 ratio with a caliper width of 0.2, and discarding of cases or controls was allowed during the process. Standard differences (*D*) were calculated to evaluate the balance of the distribution of the baseline characteristics between the two groups before and after PS matching. *D <*0.1 was used as the threshold to indicate a negligible difference in the mean or prevalence of a covariate between exposure groups. The matching was carried out with the MatchIt package.

Multivariate analysis (logistical or log-binomial regression) was also performed to assess the association between different insemination methods and CLBR in both unmatched and matched cohorts, after adjusting for important covariates previously mentioned.

To further evaluate the impact on embryo development, we further investigated the effect of short-term insemination on blastocyst development in a subgroup of patients with all their cleavage embryos extended culture. Potential interactions between short-term insemination and blastocyst culture were estimated in a multivariate model adjusted for previously mentioned covariates on both matched and unmatched cohorts. The coefficient of the interaction term was interpreted as a ratio of OR (ORR), which demonstrated a deviation from the product of treatment subgroup effects to demonstrate a potential effect modification.

Continuous variables were represented as median [first quartile, third quartile], while categorical variables were represented as *N* (percentage). The Wilcoxon test was used for continuous variables, and the chi-square test was used for categorical variables. *P <*0.05 was considered to be statistically significant.

All analyses were based on the R software (R Core Team (2022). R: A language and environment for statistical computing).

## Results

A total of 2,392 IVF cycles with one or two oocytes retrieved were analyzed in this study, including 2,057 cycles of short-term insemination and 335 cycles of overnight insemination. After PS matching, 581 cycles in the short-term insemination group were matched with 312 cycles in the overnight insemination group. The basal characteristics before and after PS matching are shown in **Table 1**. Before matching, there were significant differences between the two groups in female age, ovarian stimulation protocol, GN starting dose, and percentage of cycles with total day 3 embryos extended culture (*D* > 0.1). After matching, the two groups were similar in baseline characteristics.

**Table 1:** Basic characteristics before and after PS matching for OPU cycles.

Variables	Before matching		*D*	After matching		*D*
	Short-term	Overnight		Short-term	Overnight	
	(*N* = 2,057)	(*N* = 335)		(*N* = 581)	(*N* = 312)	
Female age, years	35.0 [31.0, 39.0]	36.0 [32.0,39.0]	0.1585	37.0 [32.0, 40.0]	36.0 [32.0, 39.0]	0.0033
History of spontaneous miscarriage						
0	1726 (83.9%)	288 (86.0%)	0.0206	487 (83.8%)	268 (85.9%)	0.0208
1	275 (13.4%)	43 (12.8%)	−0.0053	84 (14.5%)	40 (12.8%)	−0.0176
2	39 (1.9%)	4 (1.2%)	−0.007	10 (1.7%)	4 (1.3%)	−0.0032
≧3	17 (0.8%)	0 (0%)	−0.0083			
Parity ≧ 1 (%)	623 (30.3%)	120 (35.8%)	0.0553	207 (35.6%)	110 (35.3%)	−0.0064
OPU order						
1	1,303 (63.3%)	224 (66.9%)	0.0352	375 (64.5%)	203 (65.1%)	0.0096
2	492 (23.9%)	66 (19.7%)	−0.0422	127 (21.9%)	65 (20.8%)	−0.0064
≧3	262 (12.7%)	45 (13.4%)	0.007	79 (13.6%)	44 (14.1%)	−0.0032
Endometriosis (%)	273 (13.3%)	43 (12.8%)	−0.0044	69 (11.9%)	41 (13.1%)	0.0144
PCOs (%)	42 (2.0%)	4 (1.2%)	−0.0085	10 (1.7%)	3 (1.0%)	−0.008
Duration of infertility, years	4.00 [2.00, 6.20]	3.00 [1.73, 5.00]	−0.3418	3.00 [1.80, 5.00]	3.10 [1.80, 5.01]	−0.0351
Female BMI, kg/cm^2^	21.8 [20.1, 23.2]	21.9 [20.3, 23.4]	0.1112	22.0 [20.5, 23.2]	22.0 [20.3, 23.4]	−0.0029
Basal FSH, IU/L	8.87 [7.06, 11.7]	9.49 [7.56, 12.0]	0.035	9.06 [7.11, 11.7]	9.46 [7.52, 11.8]	0.0647
Basal LH, IU/L	3.87 [2.81, 5.42]	3.95 [2.90, 5.51]	0.0603	3.88 [2.70, 5.39]	3.95 [2.91, 5.44]	0.0123
Antral follicle count	5.00 [2.00, 7.00]	3.00 [1.00, 6.00]	−0.3813	4.00 [1.00, 6.00]	4.00 [1.00, 6.00]	−0.0844
Male age, years	36.0 [32.0, 40.0]	36.0 [32.0, 40.0]	0.039	36.0 [32.0, 41.0]	36.0 [32.0, 40.0]	−0.0107
Male BMI, kg/cm^2^	24.0 [21.8, 26.0]	24.0 [22.1, 26.4]	0.0756	24.1 [22.2, 26.2]	24.1 [22.1, 26.4]	−0.0255
Sperm normal morphology rate (%)	6.00 [4.0, 9.0]	6.00 [4.00, 10.0]	−0.0049	6.00 [4.0, 9.0]	6.00 [4.00, 10.0]	0.0241
Total motile sperm number (×10^6^)	54.6 [27.1, 80.3]	49.2 [24.5, 78.4]	−0.248	48.1 [24.0, 87.2]	51.7 [24.5, 84.9]	−0.0498
Ovarian stimulation protocols (%)						
Luteal phase	86 (4.2%)	22 (6.6%)	0.0239	32 (5.5%)	19 (6.1%)	0.0048
Mild stimulation	119 (5.8%)	12 (3.6%)	−0.022	26 (4.5%)	12 (3.8%)	−0.0032
GnRH antagonist	969 (47.1%)	201 (60.0%)	0.1289	344 (59.2%)	182 (58.3%)	−0.016
GnRH agonist	698 (33.9%)	81 (24.2%)	−0.0975	151 (26.0%)	80 (25.6%)	0.0016
Other protocols	12 (0.6%)	3 (0.9%)	0.0031	5 (0.9%)	3 (1.0%)	<0.001
Natural cycle	173 (8.4%)	16 (4.8%)	−0.0363	23 (4.0%)	16 (5.1%)	0.0128
GN starting dose, IU	225 [225, 225]	225 [225, 225]	0.2239	225 [225, 225]	225 [225, 225]	−0.0233
Oocyte yield			0.0166			−0.0192
1	943 (45.8%)	148 (44.2%)		250 (43.0%)	137 (43.9%)	
2	1114 (54.2%)	187 (55.8%)		331 (57.0%)	175 (56.1%)	
Cycles with all embryos extended culture (%)	111 (5.4%)	76 (22.7%)	0.1729	95 (16.4%)	54 (17.3%)	−0.0304

Data are presented as median [first quartile, third quartile] for continuous variables and *n* (percentage) for categorical variables. *D: standardized difference. The absolute value of *D* is less than 0.1; cohorts can be considered to be balanced with respect to the demographics being assessed.

OPU, oocyte pick-up; PCOS, polycystic ovarian syndrome; BMI, body mass index; FSH, follicle-stimulating hormone; LH, luteinizing hormone; GnRH, gonadotropin-releasing hormone; GN, gonadotropin.

**Table 2** shows the outcomes of the short-term insemination group and the overnight insemination group before and after PS matching. After matching, no significant difference was observed between the two groups in terms of complete total fertilization failure (TFF) cycle rate, mature oocyte number, fertilization oocyte number, available embryo number, high-quality embryo number, and CLBRs between the two groups after matching (*P* > 0.05). However, after PS matching, the canceled ET cycle rate, cycles with no embryo rate, freeze-all cycle rate, and MPN rate in the short-term insemination group were significantly lower than those in the overnight insemination group (*P* < 0.05). Additionally, the short-term insemination group had a significantly higher number of normal fertilization, normal fertilization rate, and cleavage 2PN embryo rate compared to the overnight insemination group (*P* < 0.05).

**Table 2:** Clinical outcomes before and after PS matching for OPU cycles.

Variables	Before matching		*P*-value	After matching		*P*-value
	Short-term	Overnight		Short-term	Overnight	
	(*N* = 2,057)	(*N* = 335)		(*N* = 581)	(*N* = 312)	
Canceled ET cycles (%)	715 (34.8%)	201 (60.0%)	<0.001	217 (37.3%)	182(58.3%)	<0.001
Cycles got no embryo (%)	550 (26.7%)	139 (41.5%)	<0.001	152 (26.2%)	131(42.0%)	<0.001
Freeze-all cycle (%)	165 (8.0%)	62 (18.5%)	<0.001	65 (11.2%)	51 (16.3%)	0.037
TFF cycle (%)	184 (8.9%)	28 (8.4%)	0.805	43 (7.4%)	28 (9.0%)	0.485
Mature oocyte number	1.00[1.00, 2.00]	1.00[1.00, 2.00]	0.168	1.00[1.00, 2.00]	1.00[1.00, 2.00]	0.968
Fertilization oocyte number	1.00 [1.00, 2.00]	1.00 [1.00, 2.00]	0.625	1.00 [1.00, 2.00]	1.00[1.00, 2.00]	0.387
Normal fertilization-embryo number	1.00 [1.00, 1.00]	1.00 [0, 2.00]	0.332	1.00 [1.00, 2.00]	1.00 [0, 2.00]	0.008
Normal fertilization rate (%)	100 [50.0, 100]	100 [0, 100]	0.181	100 [50.0, 100]	50.0 [0, 100]	0.003
MPN rate (%)	0 [0, 0]	0 [0, 0]	0.0567	0 [0, 0]	0 [0, 50.0]	0.0011
Cleavage 2PN embryo rate (%)	100 [0, 100]	100 [0, 100]	0.0218	100 [100, 100]	100 [0, 100]	<0.001
Available embryo number	1.00 [0, 1.00]	1.00 [0, 1.00]	<0.001	1.00 [0, 1.00]	1.00 [0, 1.00]	0.912
High-quality embryo number	0 [0, 1.00]	0 [0, 1.00]	<0.001	0 [0, 1.00]	0 [0, 1.00]	0.79
CLB rate (%)	435 (21.1%)	59 (17.6%)	0.159	120 (20.7%)	56 (17.9%)	0.378

Data are presented as median [first quartile, third quartile] and mean (SD) for continuous variables and *n* (percentage) for categorical variables. Some parameters (such as mature oocyte number) were difficult to distinguish the differences between the two groups in this table, so we presented continuous variables as median [first quartile, third quartile] and mean (SD) in **Supplementary Table S3**.

TFF, total fertilization failure; PN, pronuclei; MPN, multiple pronuclei; CLB, cumulative live birth.

We also performed a multivariate generalized estimating equation (GEE) analysis, with adjustments made for important covariates and potential confounding factors, and the results of the analysis in **Supplementary Table S1** further showed that compared to overnight insemination, short-term insemination in IVF cycles did not affect the CLBRs both in the unmatched cohort (OR: 0.79; 95% CI: 0.57–1.09) and matched cohort (OR: 0.85; 95% CI: 0.58–1.22).

In this study, a total of 188 cycles were analyzed for blastocyst culture subgroup analysis, including 111 cycles in the short-term insemination group and 76 cycles in the overnight insemination group. After matching, 95 cycles in the short-term insemination group were paired with 54 cycles in the overnight insemination group. The basic characteristics before and after PS matching are shown in **Supplementary Table S2**. Before matching, significant differences were observed between the two groups in OPU order, duration of infertility, AFC, TMC, and GN starting dose (*P* < 0.05). After matching, all baseline characteristics between the two groups were similar.

**Table 3** shows the outcomes of the short-term insemination group and the overnight insemination group in the blastocyst culture cycles. After matching, there was no significant difference between the two groups in the no embryo transfer cycle rate, freeze-all cycle rate, mature oocyte number, fertilization oocyte number, 2PN embryo number, available embryo number, high-quality embryo number, extended culture embryo number on day 3, blastocyst formation rate, and blastocyst culture outcome (P > 0.05). The cumulative live birth rate of the short-term insemination group was higher than that of the overnight insemination group, but this difference was not statistically significant (24.2% vs. 11.1%, *P* > 0.05). Notably, the ET cancellation rate in the short-term insemination group was significantly lower than that in the overnight insemination group (77.9% vs. 94.4%, P < 0.05). The results of blastocyst culture indicated that on the 5th day, the on-time blastocyst formation rate of the short-term insemination group was higher than that of the overnight insemination group, though this difference also lacked statistical significance (40.0% vs. 24.1%, P > 0.05). The delayed blastocyst formation rate in the short-term insemination group was lower than that in the overnight insemination group, with no statistical significance observed either (18.9% vs. 31.5%, P > 0.05).

**Table 3:** Clinical outcomes before and after PS matching for blastocyst culture cycles.

Variables	Before matching		*P*-value	After matching		*P*-value
	Short-term	Overnight		Short-term	Overnight	
	(*N* = 111)	(*N* = 76)		(*N* = 95)	(*N* = 54)	
Cancelled ET cycle rate (%)	83 (74.8%)	70 (92.1%)	0.0047	74 (77.9%)	51 (94.4%)	0.016
No embryo transfer cycle rate (%)	44 (39.6%)	34 (44.7%)	0.587	40 (42.1%)	26 (48.1%)	0.588
Freeze all cycle rate (%)	39 (35.1%)	36 (47.4%)	0.127	34 (35.8%)	25 (46.3%)	0.277
Mature oocyte number, mean (SD)	2.00 [1.00, 2.00]	2.00 [1.00, 2.00]	0.938	2.00 [1.00, 2.00]	2.00[1.00,2.00]	0.663
Fertilization oocyte number	2.00 [1.00, 2.00]	1.00 [1.00, 2.00]	0.284	2.00 [1.00, 2.00]	1.50 [1.00, 2.00]	0.855
2PN embryo number	1.00 [1.00, 2.00]	1.00 [1.00, 2.00]	0.901	1.00 [1.00, 2.00]	1.00 [1.00, 2.00]	0.85
Available embryo number	1.00 [1.00, 2.00]	1.00 [1.00, 2.00]	0.81	1.00 [1.00, 2.00]	1.00 [1.00, 2.00]	0.981
High-quality embryo number	1.00 [0, 1.00]	0 [0, 1.00]	0.269	1.00 [0, 1.00]	0 [0, 1.00]	0.094
Extended culture embryo number on day 3	1.00 [1.0, 2.0]	1.00 [1.00,2.00]	0.292	1.00 [1.00,2.00]	1.00 [1.00,2.00]	0.633
Blastocyst formation rate, (%)	50.0 [0,100]	50.0 [0,100]	0.636	50.0 [0,100]	50.0 [0,100]	0.944
Blastocyst culture outcome			0.083			0.084
No formation rate, (%)	43 (38.7%)	31 (40.8%)		39 (41.1%)	24 (44.4%)	
On-time formation rate, (%)	46 (41.4%)	21 (27.6%)		38 (40.0%)	13 (24.1%)	
Delay formation rate, (%)	22 (19.8%)	24 (31.6%)		18 (18.9%)	17 (31.5%)	
CLB rate, (%)	31 (27.9%)	8 (10.5%)	0.007	23 (24.2%)	6 (11.1%)	0.084

Data are presented as median [first quartile, third quartile] and mean (SD) for continuous variables and *n* (percentage) for categorical variables. Some parameters (such as mature oocyte number) were difficult to distinguish the differences between the two groups in this table, so we presented continuous variables as median [first quartile, third quartile] and mean (SD) in **Supplementary Table S4**.

ET, embryo transfer; PN, pronuclei; CLB, cumulative live birth.

When patients were subgrouped based on whether blastocyst culture was performed, regardless of whether matching was performed, the association between the short-term insemination group and the cumulative live birth rate was only observed in the blastocyst culture cycle. After adjusting the potential confounding factors in the blastocyst culture cycle, the relative risks (RRs) for CLBR when comparing overnight insemination to short-term insemination were 0.40 (95% CI: 0.18–0.88) and 0.39 (95% CI: 0.18–0.84) in the unmatched and matched cohorts, respectively. In the interaction analyses, OR ratios in the overall and matched cohorts were 0.3 (95% CI: 0.11, 0.74) and 0.36 (95% CI: 0.11, 1.04), respectively. The *P*-values of interaction for the unmatched and matched cohorts were 0.012 and 0.07, respectively, and the results are shown in **Table 4**.

**Table 4:** Interaction between the extended culture and short-term insemination.

Groups	Insemination	*N*	Live birth rate (%)	Crude RR (95% CI)	Adjusted RR (95% CI)^a^	Adjusted OR (95% CI)	OR ratio (95% CI)	RR ratio (95% CI)
Overall cohort								
No extended culture	Short-term	1,946	404 (20.8%)	Ref	Ref	Ref	Ref	ref
	Overnight	259	51 (19.7%)	0.95 (0.73, 1.23)	0.98 (0.76, 1.25)	0.95 (0.67, 1.33)	Ref	ref
Extended culture	Short-term	111	31 (27.9%)	Ref	Ref	Ref	Ref	ref
	Overnight	76	8 (10.5%)	0.38 (0.18, 0.77)	0.40 (0.18, 0.88)	0.32 (0.11, 0.87)	0.3 (0.11, 0.74)	0.41 (0.19,0.86)
Matched cohort								
No extended culture	Short-term	486	97 (20.0%)	Ref	Ref	Ref	Ref	Ref
	Overnight	258	50 (19.4%)	0.97 (0.72, 1.32)	1.02 (0.76, 1.37)	0.98 (0.66, 1.48)	Ref	Ref
Extended culture	Short-term	95	23 (24.2%)	Ref	Ref	Ref	Ref	Ref
	Overnight	54	6 (11.1%)	0.46 (0.2, 1.06)	0.39 (0.18, 0.84)	0.33 (0.11, 1.03)	0.36 (0.11, 1.04)	0.50 (0.21, 1.2)

a Adjusted for female age, female BMI, female basal FSH, LH, AFC, previous maternal history, PCOS, endometriosis, OPU order, male BMI, male age, sperm normal morphology rate, total motile sperm number, stimulation protocol, oocyte yield, and total day 3 embryo extended culture cycle rate.

## Discussion

The present study showed that compared with overnight insemination, short-term insemination got similar cumulative live birth rates in poor responders with one or two oocytes retrieved. However, poor responders receiving overnight insemination got higher rates of no embryo for transfer, TFF, and MPN. Additionally, subgroup analysis suggested that short-term insemination may favor the extended *in vitro* culture in terms of blastocyst development and cumulative live birth rates in cycles with extended blastocyst culture.

Only a few previous studies on short-term insemination have evaluated cumulative live birth rates. The RCT reported by Chen et al. reported similar live birth rates per intent-to-treat cycle in patients receiving short-term insemination compared to overnight insemination following fresh embryo transfer (4). They also carried out a 1-year follow-up to evaluate the cumulative live birth; however, the duration of follow-up is shorter than the 2 years proposed by Maheshwari et al. (24). More recently, Jiang et al. reported that the cumulative live birth rate following short-term insemination (3 h) combined with early rescue ICSI was comparable with that of patients receiving ICSI (11). In their study, the cumulative live birth rate was well-defined; however, the inclusion of patients with ICSI/RICSI indications may have introduced bias to the study population. In contrast, Sun et al. suggested that short-term insemination (5 h) can increase the cumulative live birth rate but with a lower fertilization rate compared with overnight IVF (25). In the present study, we found no significant difference in cumulative live birth between short-term insemination and overnight insemination in patients with only one or two oocytes retrieved, but the tendency may favor short-term insemination. It could be explained by the overall low fecundity of these patients, as CLBR is sensitive to oocyte yield (26). A lower absolute difference may need a larger sample size to verify.

In this study, it is also notable that the rate of no embryo transfer cycle in the short-term insemination group was lower than that in the overnight insemination group. Cycles resulting in no embryo are not only relevant to CLBR but also bring a heavy economic and emotional burden to patients (27). Considerable emotional suffering was recognized after no embryo transfer, and they may need early feedback concerning reasons for failure and future alternatives (28). Since approximately 9%–24% of patients receiving IVF treatment are poor responders (29) and this population is more likely to encounter cycle cancellation and no embryo transfer (30, 31), the cancellation due to the absence of available embryos may be a major concern for these patients. Additionally, the significant difference in transfer cancellation suggested that an unelectable bias would be found when only fresh transfers were evaluated in a study design (32).

We also noted that patients undergoing short-term insemination may have favorable laboratory parameters, such as fertilization, embryo cleavage, and blastocyst culture, even in poor responders. This finding may further support the hypothesis that short-term insemination reduces ROS stress during culture (5, 33). Importantly, the subgroup analyses and interaction also suggested a potential effect of insemination duration on extended embryo culture. Extending embryo culture to the blastocyst stage represents an important aspect of contemporary clinical practice, as it offers a better evaluation of the developmental potential of embryos, provides a means of embryo election, reduces the number of embryos transferred, and enables patients to obtain pregnancy faster (34, 35). For patients with low oocyte yield, blastocyst culture and transfer may also serve as a viable clinical strategy for poor responders (36). However, the cycle cancellation due to no blastocyst formation may increase with low oocyte yield. Although no effect on overall blastocyst formation rates was observed in our data, overnight insemination was associated with more delayed blastocysts, which may be detrimental to subsequent transfer. If the embryo transfer strategy favors blastocyst, a more profound effect of short-term insemination would be expected.

MPN rates following fertilization may also be a significant concern of short-term insemination (37). Many studies suggested an increased MPN rate in short-term insemination cycles, several of which were associated with RICSI (38). Notably, in studies reporting cycles with short-term insemination only, the conclusions were also inconsistent (17, 39, 40). A retrospective study showing that the removal of cumulus cells after short-term insemination (5 h) significantly reduced the MPN rates following fertilization may support our finding (41). These previous studies vary in the co-incubation time of insemination. It has been shown that the timing of early removal of cumulus cells had a significant effect on the rate of MPN; removing cumulus cells earlier than 5 h may increase MPN rates (17). Therefore, the variation in co-incubation time may contribute to the inconsistency, and an optimal timing of cumulus cell removal remains to be determined.

Another factor that may influence fertilization outcomes is the sperm concentration used for insemination. Studies have shown that the use of overnight insemination at higher sperm concentration leads to an increase in polyspermy fertilization rate both in animals and humans (8, 16, 42). In previous studies, a wide range of sperm concentrations have been employed (16), and a similar sperm concentration was used for both short-term and overnight (38–41, 43). These factors might also affect the interpretation of the results.

Some limitations of this study should be considered. First, it is a retrospective cohort study that is affected by unmeasured or unknown confounders or biases. Although a significant difference was observed between the subgroups of patients receiving blastocyst culture and those who did not, the sample sizes of the subgroups are limited, and the blastocyst culture subgroup is potentially biased. Additionally, cautions should also be taken because this study was carried out in a single center, solely based on the Cook culture system. Since Cook Medical stopped producing the culture media, whether the results could be generalized to other culture environments is unknown.

## Conclusions

While short-term insemination has been suggested as an effective and safe alternative to overnight insemination in numerous studies, it still lacks well-defined indications. For clinics carrying out short-term insemination, whether to provide short-term insemination for all IVF-indicated patients may be an intriguing issue. Some clinicians may exclude patients with fewer than three oocytes retrieved from their short-term insemination cohort (44). Our data showed that for patients with low oocyte yield, short-term insemination may not harm cumulative live birth outcomes and may have a potential benefit on embryo culture. The workflow of the laboratory and the acceptance of the patients might be the primary concerns for the decision.

## Data Availability

The raw data supporting the conclusions of this article will be made available by the authors, without undue reservation.
