# Supplementary material for: Short-term vs. overnight insemination: which one is better for patients with only one or two oocytes retrieved in IVF cycles?
Source: Front Endocrinol (Lausanne). 2026 Feb 3;17:1647862. doi: 10.3389/fendo.2026.1647862 (PMC12909161; doi:10.3389/fendo.2026.1647862)
Supplement: Supplementary file 1 [file DataSheet1.docx]

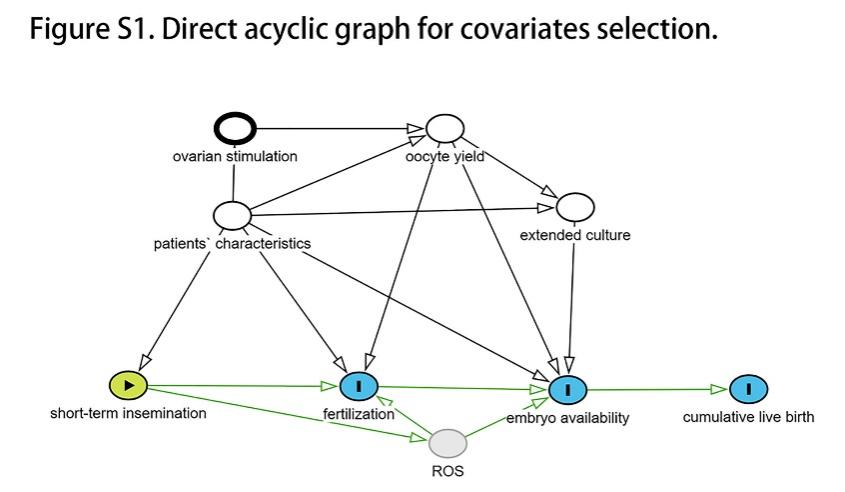


Figure S1. Direct acyclic graph for covariates selection.

Table S 1. Summary of logistic regression analysis results.

| Characteristic | Before Matching | | p-value | After Matching | | p-value |
| --- | --- | --- | --- | --- | --- | --- |
|  | OR*^1^* | 95% CI*^1^* |  | OR*^1^* | 95% CI*^1^* |  |
| Factor (Overnight) |  |  |  |  |  |  |
| overnight | ref | ref |  | ref | ref |  |
| Short-term | 0.79 | 0.57, 1.09 | 0.2 | 0.85 | 0.58, 1.22 | 0.4 |
| Female's age, year | 0.95 | 0.92, 0.98 | 0.004 | 0.97 | 0.91, 1.02 | 0.3 |
| History of spontaneous miscarriage | | |  |  |  |  |
| 0 | ref | ref |  | ref | ref |  |
| 1 | 1.03 | 0.75, 1.40 | 0.9 | 1.29 | 0.77, 2.11 | 0.3 |
| 2 | 0.81 | 0.32, 1.80 | 0.6 | 1.59 | 0.33, 5.86 | 0.5 |
| ≧3 | 0.21 | 0.01, 1.07 | 0.13 |  |  |  |
| Parity |  |  |  |  |  |  |
| 0 | ref | ref |  | ref | ref |  |
| ≧1 | 0.97 | 0.75, 1.25 | 0.8 | 1.18 | 0.78, 1.78 | 0.4 |
| OPU order |  |  |  |  |  |  |
| 1 | ref | ref |  | ref | ref |  |
| 2 | 1.32 | 1.03, 1.69 | 0.028 | 1.33 | 0.87, 2.02 | 0.2 |
| ≧3 | 0.85 | 0.58, 1.22 | 0.4 | 0.6 | 0.30, 1.12 | 0.12 |
| Endometriosis | 0.86 | 0.63, 1.18 | 0.4 | 0.76 | 0.43, 1.31 | 0.3 |
| PCOS | 1.03 | 0.49, 2.14 | >0.9 | 0.85 | 0.19, 3.30 | 0.8 |
| Duration of infertility | 0.96 | 0.92, 0.99 | 0.009 | 1 | 0.94, 1.06 | >0.9 |
| female BMI | 1.03 | 0.98, 1.07 | 0.3 | 1.06 | 0.97, 1.16 | 0.2 |
| Basal FSH | 1 | 1.00, 1.03 | 0.5 | 0.98 | 0.93, 1.02 | 0.4 |
| Basal LH | 1.04 | 0.99, 1.08 | 0.076 | 1.02 | 0.95, 1.09 | 0.6 |
| AFC | 1 | 0.97, 1.03 | >0.9 | 1 | 0.95, 1.05 | >0.9 |
| Male's age | 0.98 | 0.96, 1.01 | 0.3 | 0.94 | 0.90, 0.99 | 0.016 |
| Male BMI | 0.98 | 0.95, 1.01 | 0.2 | 0.97 | 0.92, 1.02 | 0.3 |
| Sperm normal morphology rate | 0.98 | 0.96, 1.01 | 0.2 | 0.97 | 0.93, 1.01 | 0.11 |
| Total motile sperm number | 1 | 1.00, 1.00 | 0.055 | 1 | 1.00, 1.00 | 0.2 |
| Stimulation protocols |  |  |  |  |  |  |
| Luteal phase | ref | ref |  | ref | ref |  |
| Mild stimulation protocol | 1.91 | 0.85, 4.49 | 0.13 | 1.23 | 0.29, 5.16 | 0.8 |
| GnRH antagonist | 2.19 | 1.14, 4.60 | 0.027 | 1.89 | 0.73, 5.93 | 0.2 |
| GnRH agonist | 2.76 | 1.42, 5.84 | 0.005 | 2.35 | 0.87, 7.56 | 0.11 |
| Other protocols | 1.88 | 0.36, 7.62 | 0.4 | 3.58 | 0.41, 23.6 | 0.2 |
| Natural cycle | 0.43 | 0.15, 1.28 | 0.13 | 0.4 | 0.04, 3.30 | 0.4 |
| GN starting dose, IU | 1 | 0.99, 1.00 | 0.03 | 1 | 0.99, 1.00 | 0.4 |
| Oocyte yield | 1.89 | 1.51, 2.37 | <0.001 | 1.88 | 1.29, 2.77 | 0.001 |
| All day 3 embryo extended culture cycles rate, % | 1.22 | 0.81, 1.80 | 0.3 | 1.07 | 0.66, 1.72 | 0.8 |

1 OR = Odds Ratio; CI = Confidence Interval;

Table S2. Basic characteristics and embryo development for blastocyst culture cycles.

|  | Before Matching | | P-value | After Matching | | P-value |
| --- | --- | --- | --- | --- | --- | --- |
|  | Short-term | Overnight |  | Short-term | Overnight |  |
|  | (N=111) | (N=76) |  | (N=95) | (N=54) |  |
| Female age, year | 36.0 [32.5,40.0] | 36.5 [32.0,41.0] | 0.947 | 36.0 [33.0,40.0] | 36.5 [32.0,40.5] | 0.814 |
| History of spontaneous miscarriage | | | 0.279 |  |  | 0.521 |
| 0 | 84 (75.7%) | 65 (85.5%) |  | 75 (78.9%) | 46 (85.2%) |  |
| 1 | 22 (19.8%) | 10 (13.2%) |  | 19 (20.0%) | 7 (13.0%) |  |
| 2 | 2 (1.8%) | 1 (1.3%) |  | 1 (1.1%) | 1 (1.9%) |  |
| ≧3 | 3 (2.7%) | 0 (0%) |  |  |  |  |
| Parity ≧1 (%) | 38 (34.2%) | 25 (32.9%) | 0.974 | 35 (36.8%) | 15 (27.8%) | 0.344 |
| OPU order | |  | 0.0026 |  |  | 0.15 |
| 1 | 48 (43.2%) | 52 (68.4%) |  | 41 (43.2%) | 32 (59.3%) |  |
| 2 | 34 (30.6%) | 15 (19.7%) |  | 31 (32.6%) | 14 (25.9%) |  |
| ≧3 | 29 (26.1%) | 9 (11.8%) |  | 23 (24.2%) | 8 (14.8%) |  |
| Endometriosis (%) | 11 (9.9%) | 11 (14.5%) | 0.471 | 10 (10.5%) | 9 (16.7%) | 0.41 |
| PCOS (%) | 1 (0.9%) | 0 (0%) | >0.99 | 1 (1.1%) | 0 (0%) | >0.99 |
| Duration of infertility, year | 3.40 [1.95,6.50] | 2.80 [1.45,4.20] | 0.0461 | 3.00 [1.95,5.90] | 3.05 [1.80,5.03] | 0.458 |
| Female BMI, kg/cm2 | 22.2 [20.7,23.2] | 22.4 [20.2,23.5] | 0.569 | 22.3 [20.7,23.3] | 22.9 [20.0,23.6] | 0.568 |
| Basal FSH, IU/l | 9.54 [7.50,12.7] | 9.65 [7.78,12.2] | 0.672 | 9.55 [7.50,12.9] | 9.46 [7.59,11.3] | 0.537 |
| Basal LH, IU/l | 3.80 [2.64,5.53] | 3.44 [2.37,5.54] | 0.416 | 3.67 [2.59,5.53] | 3.12 [2.17,4.97] | 0.267 |
| Antral follicle count | 5.00 [3.00,7.00] | 3.00 [0,5.25] | <0.001 | 4.00 [3.00,6.50] | 4.00 [2.00,7.75] | 0.611 |
| Male age, year | 38.0 [33.0,41.0] | 35.0 [32.0,40.0] | 0.347 | 38.0 [32.0,41.0] | 35.0 [32.0,40.0] | 0.621 |
| Male BMI, kg/cm2 | 24.2 [22.1,25.9] | 24.4 [22.4,26.5] | 0.501 | 24.2 [22.1,26.0] | 24.6 [22.2,26.6] | 0.67 |
| Sperm normal morphology rate (%) | 6.00 [4.00,9.00] | 6.25 [4.38,10.1] | 0.266 | 6.00 [4.00,9.00] | 6.00 [4.13,10.0] | 0.343 |
| Total motile sperm number （x106） | 64.9 [34.9,105] | 49.0 [28.1,77.0] | 0.0412 | 58.2 [32.3,101] | 64.3 [35.9,89.6] | 0.978 |
| Ovarian stimulation protocols (%) | | | 0.098 |  |  | 0.292 |
| Luteal phase | 13 (11.7%) | 5 (6.6%) |  | 12 (12.6%) | 2 (3.7%) |  |
| Mild stimulation | 3 (2.7%) | 4 (5.3%) |  | 2 (2.1%) | 4 (7.4%) |  |
| GnRH antagonist | 56 (50.5%) | 52 (68.4%) |  | 53 (55.8%) | 33 (61.1%) |  |
| GnRH agonist | 29 (26.1%) | 9 (11.8%) |  | 20 (21.1%) | 9 (16.7%) |  |
| Other protocols | 1 (0.9%) | 1 (1.3%) |  | 1 (1.1%) | 1 (1.9%) |  |
| Natural cycle | 9 (8.1%) | 5 (6.6%) |  | 7 (7.4%) | 5 (9.3%) |  |
| GN starting dose, IU | 225  [188,225] | 225  [225,225] | 0.00157 | 225  [225,225] | 225 [225,225] | 0.0509 |
| Oocyte yield | |  | 0.95 |  |  | 0.833 |
| 1 | 45 (40.5%) | 32 (42.1%) |  | 40 (42.1%) | 21 (38.9%) |  |
| 2 | 66 (59.5%) | 44 (57.9%) |  | 55 (57.9%) | 33 (61.1%) |  |

Data were presented as median [first quartile, third quartile] for continuous variables and N (percentage) for categorical variables. OPU, oocyte pick up; PCOS, polycystic ovarian syndrome; GNRH, gonadotropin; BMI, body mass index; FSH, follicle-stimulating hormone; LH, luteinizing hormone; GN, gonadotropin.

**Table S3**. Clinical outcomes before and after PS matching for OPU cycles

|  | Before Matching | |  | After Matching | |  |
| --- | --- | --- | --- | --- | --- | --- |
| Variables | Short-term | Overnight | P-value | Short-term | Overnight | P-value |
|  | (N=2057) | (N=335) |  | (N=581) | (N=312) |  |
| Canceled ET cycles (%) | 715(34.8%) | 201(60.0%) | <0.001 | 217 (37.3%) | 182(58.3%) | <0.001 |
| Cycles got no embryo (%) | 550(26.7%) | 139 (41.5%) | <0.001 | 152 (26.2%) | 131(42.0%) | <0.001 |
| Freeze-all cycle (%) | 165 (8.0%) | 62 (18.5%) | <0.001 | 65 (11.2%) | 51 (16.3%) | 0.037 |
| TFF cycle (%) | 184 (8.9%) | 28 (8.4%) | 0.805 | 43 (7.4%) | 28 (9.0%) | 0.485 |
| Mature oocyte number |  |  |  |  |  |  |
| Median [Q1, Q3]  Mean (SD) | 1.00[1.00,2.00]  1.41(0.582) | 1.00[1.00,2.00]  1.46 (0.567) | 0.168 | 1.00[1.00,2.00]  1.46 (0.573) | 1.00 [1.00,2.00]  1.46 (0.571) | 0.968 |
| Fertilization oocyte number | |  |  |  |  |  |
| Median [Q1, Q3]  Mean (SD) | 1.00 [1.00,2.00]  1.32 (0.630) | 1.00 [1.00,2.00]  1.34 (0.627) | 0.625 | 1.00 [1.00,2.00]  1.38 (0.619) | 1.00[1.00,2.00]  1.34 (0.636) | 0.387 |
| Normal fertilization-embryo number | |  |  |  |  |  |
| Median [Q1, Q3]  Mean (SD) | 1.00 [1.00,1.00]  0.999 (0.704) | 1.00 [0,2.00]  0.958 (0.757) | 0.332 | 1.00 [1.00,2.00]  1.07 (0.715) | 1.00 [0,2.00]  0.929 (0.762) | 0.008 |
| Normal fertilization rate, (%) | |  |  |  |  |  |
| Median [Q1, Q3]  Mean (SD) | 100 [50.0,100] | 100 [0,100] | 0.181 | 100 [50.0,100] | 50.0 [0,100] | 0.003 |
|  | 64.0 (41.8) | 60.1 (44.1) |  | 67.1 (41.0) | 57.9 (44.4) |  |
| MPN rate, (%) |  |  |  |  |  |  |
| Median [Q1, Q3]  Mean (SD) | 0 [0,0]  13.9 (30.1) | 0 [0,0]  18.1 (34.7) | 0.0567 | 0 [0,0]  11.4 (27.3) | 0 [0,50.0]  19.4 (35.6) | 0.0011 |
| Cleavage 2PN embryo rate, (%) | |  |  |  |  |  |
| Median [Q1, Q3]  Mean (SD) | 100 [0,100]  73.6 (43.8) | 100 [0,100]  67.5 (46.8) | 0.0218 | 100 [100,100]  76.4 (42.2) | 100 [0,100]  65.4 (47.5) | <0.001 |
| Available embryo number | |  |  |  |  |  |
| Median [Q1, Q3]  Mean (SD) | 1.00 [0,1.00]  0.707 (0.736) | 1.00 [0,1.00]  0.869 (0.743) | <0.001 | 1.00 [0,1.00]  0.833 (0.757) | 1.00 [0,1.00]  0.837 (0.745) | 0.912 |
| High-quality embryo number | |  |  |  |  |  |
| Median [Q1, Q3]  Mean (SD) | 0 [0,1.00]  0.479 (0.640) | 0 [0,1.00]  0.606 (0.679) | <0.001 | 0 [0,1.00]  0.580 (0.684) | 0 [0,1.00]  0.587 (0.67) | 0.79 |
| CLB rate, (%) | 435 (21.1%) | 59 (17.6%) | 0.159 | 120 (20.7%) | 56 (17.9%) | 0.378 |

Data were presented as median [first quartile, third quartile] and mean (SD) for continuous variables and n (percentage) for categorical variables. TFF, total fertilization failure; PN, pronuclei; MPN, multi pronuclei; CLB, cumulative live birth.

**Table S4.** Clinical outcomes before and after PS matching for blastocyst culture cycles.

|  | Before Matching | |  | After Matching | |  |
| --- | --- | --- | --- | --- | --- | --- |
| Variables | Short-term | Overnight | P-value | Short-term | Overnight | P-value |
|  | (N=111) | (N=76) |  | (N=95) | (N=54) |  |
| Cancelled ET cycle rate,(%) | 83 (74.8%) | 70 (92.1%) | 0.0047 | 74 (77.9%) | 51 (94.4%) | 0.016 |
| No embryo transfer cycle rate, (%) | 44 (39.6%) | 34 (44.7%) | 0.587 | 40 (42.1%) | 26 (48.1%) | 0.588 |
| Freeze all cycle rate, (%) | 39 (35.1%) | 36 (47.4%) | 0.127 | 34 (35.8%) | 25 (46.3%) | 0.277 |
| Mature oocyte number |  |  |  |  |  |  |
| Median [Q1, Q3]  Mean (SD) | 2.00 [1.00, 2.00]  1.56 (0.499) | 2.00 [1.00, 2.00]  1.55 (0.501) | 0.938 | 2.00 [1.00, 2.00]  1.54 (0.501) | 2.00[1.00,2.00]  1.57 (0.499) | 0.663 |
| Fertilization oocyte number |  |  |  |  |  |  |
| Median [Q1, Q3]  Mean (SD) | 2.00 [1.00, 2.00]  1.54 (0.501) | 1.00 [1.00, 2.00]  1.46 (0.502) | 0.284 | 2.00 [1.00, 2.00]  1.52 (0.502) | 1.50 [1.00, 2.00]  1.50 (0.505) | 0.855 |
| 2PN embryo number |  |  |  |  |  |  |
| Median [Q1, Q3]  Mean (SD) | 1.00 [1.00, 2.00]  1.18 (0.741) | 1.00 [1.00, 2.00]  1.21 (0.639) | 0.901 | 1.00 [1.00, 2.00]  1.18 (0.729) | 1.00 [1.00, 2.00]  1.17 (0.666) | 0.85 |
| Available embryo number |  |  |  |  |  |  |
| Median [Q1, Q3]  Mean (SD) | 1.00 [1.00, 2.00]  1.12 (0.76) | 1.00 [1.00, 2.00]  1.16 (0.634) | 0.81 | 1.00 [1.00, 2.00]  1.11 (0.751) | 1.00 [1.00, 2.00]  1.11 (0.664) | 0.981 |
| High-quality embryo number | |  |  |  |  |  |
| Median [Q1, Q3]  Mean (SD) | 1.00 [0, 1.00]  0.757 (0.80) | 0 [0, 1.00]  0.618 (0.73) | 0.269 | 1.00 [0, 1.00]  0.747 (0.799) | 0 [0, 1.00]  0.519 (0.693) | 0.094 |
| Extended culture embryo number on day 3 | | |  |  |  |  |
| Median [Q1, Q3]  Mean (SD) | 1.00 [1.0, 2.0]  1.43 (0.498) | 1.00 [1.00,2.00]  1.36 (0.482) | 0.292 | 1.00 [1.00,2.00]  1.41 (0.495) | 1.00 [1.00,2.00]  1.37 (0.487) | 0.633 |
| Blastocyst formation rate, (%) | |  |  |  |  |  |
| Median [Q1, Q3]  Mean (SD) | 50.0 [0,100]  50.9 (44.7) | 50.0 [0,100]  53.9 (47.4) | 0.636 | 50.0 [0,100]  48.4 (44.6) | 50.0 [0,100]  49.1 (47.1) | 0.944 |
| Blastocyst culture outcome |  |  | 0.083 |  |  | 0.084 |
| No formation rate, (%) | 43 (38.7%) | 31 (40.8%) |  | 39 (41.1%) | 24 (44.4%) |  |
| On-time formation rate, (%) | 46 (41.4%) | 21 (27.6%) |  | 38 (40.0%) | 13 (24.1%) |  |
| Delay formation rate, (%) | 22 (19.8%) | 24 (31.6%) |  | 18 (18.9%) | 17 (31.5%) |  |
| CLB rate, (%) | 31 (27.9%) | 8 (10.5%) | 0.007 | 23 (24.2%) | 6 (11.1%) | 0.084 |

Data were presented as median [first quartile, third quartile] and mean (SD) for continuous variables and n (percentage) for categorical variables. ET, embryo transfer; PN, pronuclei; CLB, cumulative live birth.
